# Effects of Equol Supplement on Bone and Cardiovascular Parameters in Middle-Aged Japanese Women: A Prospective Observational Study

**DOI:** 10.1089/acm.2018.0050

**Published:** 2018-07-01

**Authors:** Remi Yoshikata, Khin Zay Yar Myint, Hiroaki Ohta

**Affiliations:** ^1^ Hamasite Clinic, Minato-ku, Japan.; ^2^ Tokyo Midtown Medical Center, Minato-ku, Japan.; ^3^ Sanno Medical Center, Minato-ku, Japan.

**Keywords:** equol, supplement, Japanese women, bone parameters, cardiovascular parameters

## Abstract

***Objective:*** To examine changes in the bone and cardiovascular parameters and tolerability in middle-aged Japanese women taking equol supplement for a year.

***Design:*** This was a prospective observational study.

***Subjects and Setting:*** Participants were 74 women receiving outpatient care at Hamasite Medical Clinic, Minato-ku, Tokyo, from 2013 to 2015.

***Interventions:*** Participants received per oral equol-containing supplement, 10 mg/day.

***Outcome measures:*** The primary outcome measures were percent changes in bone and cardiovascular parameters after 1 year supplementation with equol. The secondary measures included factors affecting the parameter changes and adverse effects associated with equol use for a year.

***Results:*** Reduction in arterial stiffness was observed after 12 months of equol supplement (1402.3 cm/s vs.1367.3 cm/s, *p* < 0.001). Significant reductions in respective parameters were observed in women with moderate and high risk for arteriosclerosis (median [95% confidence interval]: −3.2% [−5.79 to −0.74]; −12.65% [−18.52 to −4.28]; respectively); hypertriglyceridemia −45.53% [−70.24 to −5.58]; bone resorption risk (−15.15% [−23.71 to 1.56]; and bone fracture risk −26.68% [−76.43 to −5.99]. All 15 women with high baseline parathyroid hormone levels had achieved a median of 50% [−54.11 to −31.69] reduction from their baseline values. These associations were further confirmed in the results of multiple linear regression analysis. There were no reported adverse events or abnormal findings in the blood chemistry, Pap smear, mammography, and transvaginal ultrasound during periodic follow-ups.

***Conclusion:*** One year equol supplement was tolerable and induced improvement of certain bone and cardiovascular parameters, especially in higher risk groups. Further controlled studies are needed to explore long-term equol use for wellbeing of middle-aged women.

## Introduction

Equol is one of the active metabolites produced by the action of colonic bacteria on soy isoflavones. Dietary isoflavones are mainly made up of glycoside forms such as glycitin, daizin, and genistein, which are not readily absorbed across the enterocytes owing to their large hydrophilic structures.^1,2^ In the lumen of the small and large intestine, sugar moieties from these glycosides are hydrolyzed to form aglycones (glycitein, daidzein, and genistein) by β-glycosidases in the small intestinal brush border or colonic bacteria.^3,4^ Some aglycones are absorbed after being hydrolyzed and some remain unabsorbed in the intestinal lumen and are converted to final metabolites, such as equol and O-desmethylangolensin, by the action of colonic bacteria.^5,6^ These final metabolites are absorbed and transported into the liver via the hepatic portal circulation.

The bioavailabilities and biological activities of isoflavones are influenced by the type of diet and the ability of host colonic bacteria to metabolize them into final active metabolites.^7^ As for the metabolism of equol, certain bacteria species are required for the production of equol from daidzein,^8^ and a host genetic variant might be one of the determinants for variations in equol metabolism.^9^ Therefore, there is a great variation in the individual ability to produce equol. Although 50% of Japanese women are reported to have this equol-producing ability, only about 30% of Japanese women were found to be equol producers in our recent study.^10^ Changes in diet and the widespread use of antibiotics might have led to the reduction in the number of people who are equol producers.^11,12^

Equol resembles the chemical structure of the female hormone estrogen and it is thought to exert estrogenic actions by genomic pathways through estrogen receptors α and β in the cytoplasm and nucleus^13,14^ and by a rapid nongenomic pathway through G protein-coupled estrogen receptors.^15,16^ There is increasing evidence of its efficacy in relieving climacteric symptoms,^17,18^ and antiatherosclerotic actions.^19^ In addition, its potential action in slowing bone mineral density (BMD) loss,^20,21^ prevention of hormone-dependent cancers such as breast cancer^22,23^ and prostate^24,25^ cancers in addition to protecting against aging, diabetes, and obesity have been investigated.^26,27^

The efficacy of soy is greatly dependent on the host's ability to convert equol in the intestine by microflora. In our previous study, vascular and bone biomarkers showed favorable trends for women around the menopausal age who had the ability to produce equol.^10^ Therefore, it is postulated that the same age group of women might also benefit from dietary equol supplement. Clinical studies on equol supplements have focused on menopausal symptoms, lipid and metabolic biomarkers, and arterial stiffness,^17,28–30^ suggesting that equol supplementation has some positive effects. These studies have focused on equol's efficacy in nonproducer population. Furthermore, they are mostly short-term clinical trials and there is a need to evaluate its long-term efficacy and safety.

In this study, our primary objective was to examine the changes in bone and cardiovascular parameters among women taking equol supplement for a year. Our secondary objectives were to find out the factors affecting the parameter changes such as equol-producer status, and adverse effects associated with equol use for a year.

## Materials and Methods

### Study population

**FIG. 1.**
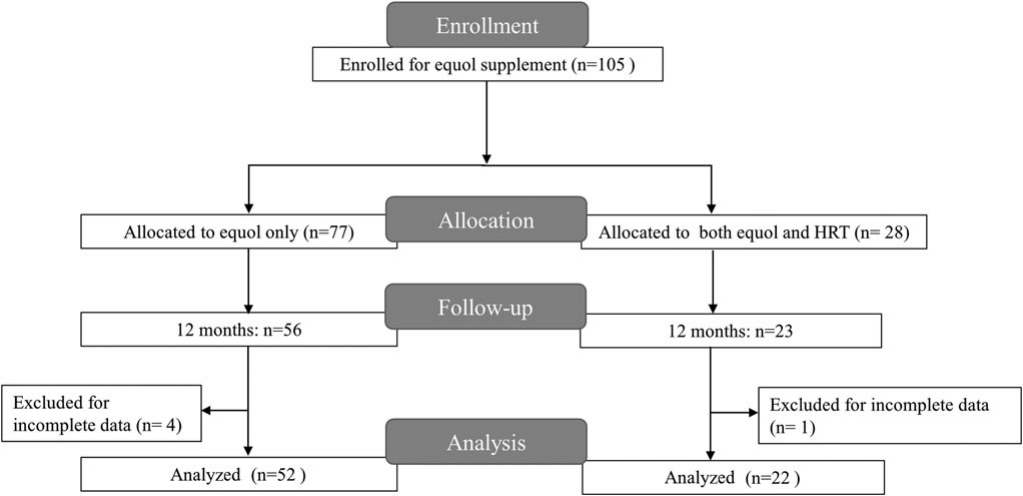
Flow chart of the observational study. HRT, hormone replacement therapy.

This prospective observational study was conducted among 105 women who were receiving outpatient care at the Hamasite Medical Clinic from 2013 to 2015 and who gave their consent to participate in the study. Among them, 74 women (44–74 years of age) completed the 12 month follow-up for blood and urine testing for bone and cardiovascular parameters (Fig. 1). Approval for the study was obtained from the Institutional Review Board of Tokyo Midtown Medical Corporation. The background characteristics of the participants were described in Table 1. Menopause was defined as no menstruation at least 12 months since last menstrual period or bilateral oophorectomy. Lifestyle-related diseases included hypertension, dyslipidemia, and diabetes. According to Japan Osteoporosis Society, osteoporosis was defined as having BMD of 70% and below in the absence of fragility fracture, or having BMD between 70% and 80% in the presence of fragility fracture, compared to average BMD values in young healthy women (Young Adult Mean: YAM).

**Table 1.** Basic Characteristics of the Participants

	*Total (*N* = 74)*
Age, years ± SD	55.14 (±7.8)
Age at menopause, years ± SD	49.33 (±4.7)
BMI, kg/m^2^	21.6 (±3.4)
Before menopause/menopause, *N*	30/44
Equol producers, *n* (%)	25^a^ (33.8%)
HRT treatment, *n* (%)	22 (29.7%)
Lifestyle-related diseases and treatment, *n* (%)	31 (41.9%)
Osteoporosis and treatment, *n* (%)	21 (28.4%)

Categorical values are shown as numbers and proportions (%) and continuous values are shown as mean (±standard deviation).

^a^ Equol-producer status was unknown for two participants.

BMI, Body–mass index; HRT, hormone replacement therapy.

### Study treatments

Participants received per oral equol-containing supplement, 10 mg/day. Among them, 22 women received combined hormone replacement therapy (HRT) as transdermal patch every 2 weeks. Decisions on equol supplement alone or combination with HRT were based on clinically evaluated facts including their most troubling symptoms, background medical history, and willingness to receive equol only or both therapies. Each equol capsule contained 98% S-equol, 2% daidzein, 0.2% glycitein, and 0.1% genistein extracted from fermented soy beans (product name: FlavoCel EQ-5; Daicel Corporation, Tokyo, Japan). One transdermal combined HRT patch contained estradiol 0.62 mg and norethistrone acetate 2.7 mg (product name: Menoaid Combipatch; Aska Pharmaceutical Co., Ltd., Tokyo, Japan). Treatments for pre-existing diseases such as lifestyle-related diseases or osteoporosis were allowed to continue.

### Determination of equol-producer status at baseline

Early morning urine samples were collected and urinary equol was measured by Healthcare Systems Co., Ltd. using Soy Check, which is an immunochromatographic strip test. The measurement method was as described in our previous study.^10^ Participants were defined as equol producers with a urinary equol level higher than 1.0 μmol/L.^31,32^

### Measurement of parameters

Anthropometric measures such as body height and weight were measured using a height weight scale (A & D Company Limited, Tokyo, Japan). Blood samples were obtained after an overnight fast to determine levels of triglycerides (TG), total cholesterol, low density lipoprotein (LDL) cholesterol, high density lipoprotein (HDL) cholesterol, intact parathyroid hormone (PTH), calcium, phosphorous, and procollagen type I propeptide (P1NP). Urinary calcium and N-telopeptide (NTX) levels were measured using early morning urine samples. To assess the degree of arterial stiffness (arteriosclerosis), branchial-ankle pulse wave velocity (baPWV) was measured using vascular ultrasound (Fukuda Denshi, Tokyo, Japan). These measurements were done at baseline and at 3 monthly follow-ups for baPWV and 6-monthly follow-ups for other parameters up to 12 months.

### Monitoring of adverse effects

Clinical signs and symptoms were monitored during monthly follow-ups. Liver and kidney function tests, and complete blood counts were done every 6 months. Pap smear, transvaginal ultrasound, and mammography were performed at baseline and after 12 months.

### Statistical analyses

Statistical analyses were performed using IBM SPSS Statistics version 19 software (IBM Japan, Minato-ku, Tokyo, Japan). Distribution of normality was assessed with the Kolmogorov–Smirnov test, box plots, and histograms. Categorical variables were expressed as numbers (*N*) and percentages (%). Continuous variables were expressed as means or medians. Baseline parameters between equol producers and nonproducers were compared using Mann–Whitney tests. Pre- and post-parameters were compared using Wilcoxon signed-rank tests. Percent changes of parameter values between the baseline and at 12 months were calculated and compared among different initial risk groups. These initial risk groups were categorized according to reference standards in Japan.

Multiple linear regression analyses were further conducted to confirm the associations between the percent changes in the parameters and their respective initial risk categories. The models were adjusted for age, equol-producing phenotype, HRT treatment; and treatments for osteoporosis in the analysis for bone parameters and lifestyle-related diseases for analysis of cardiovascular parameters. All tests were two-sided, and statistical significance was defined as *p* < 0.05.

## Results

**FIG. 2.**
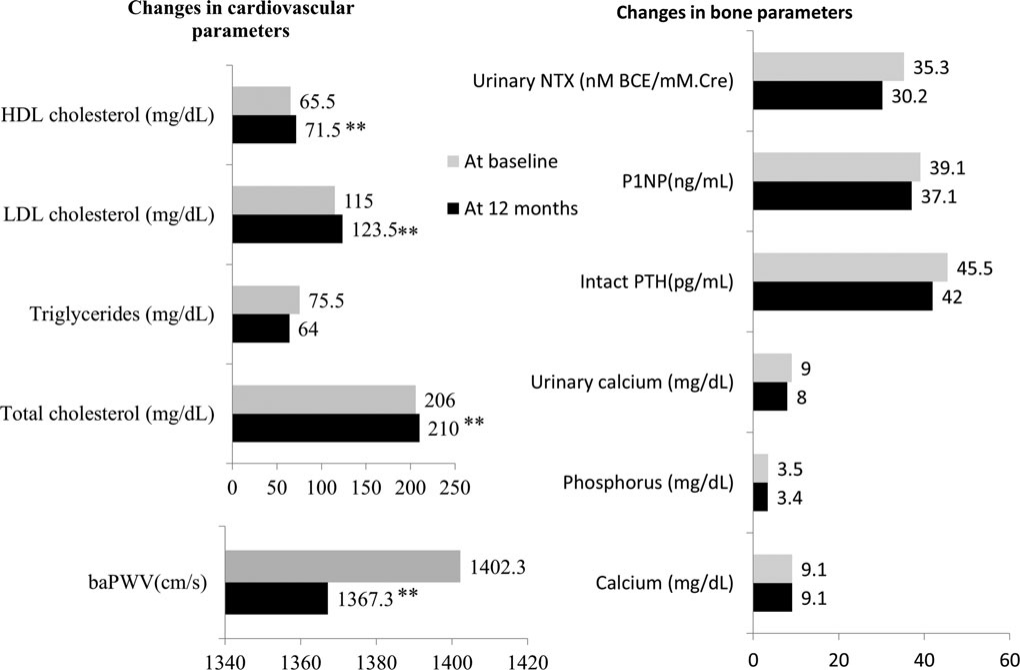
Comparison of parameters at baseline and 12 months after equol supplement (n = 74). LDL, HDL and total cholesterol levels were significantly increased. Arterial stiffness was improved significantly. Bone parameters showed a declining trend, which is not statistically significant. Values at baseline and 12th month post-treatment are shown as medians. *p-*Values are assessed by Wilcoxon signed rank test and statistically significant differences (**p < 0.01). baPWV, branchial-ankle pulse wave velocity; HDL, high density lipoprotein; LDL, low density lipoprotein cholesterol; NTX, N-telopeptide; P1NP, procollagen type I propeptide; PTH, parathyroid hormone.

Equol producers (*n* = 25) had a lower baseline baPWV (a measure of arterial stiffness) compared to nonproducers (*n* = 47), but the results has missed statistical significance (972 cm/s vs. 1656 cm/s, *p* = 0.482). Similarly, there were no significant differences between equol producers and nonproducers in lipid and bone parameters at the baseline. Significant reduction in baPWV, was observed as early as 3 months after equol supplementation in the group of women taking only equol supplement who showed up for 3-month follow-up (1429 cm/s vs. 1311 cm/s, *p* < 0.001, *n* = 45). In women who had completed 12-month follow-up, there was a significant reduction in arterial stiffness, increased levels of total cholesterol, LDL and HDL cholesterol. However, LDL/HDL ratios showed no significant change. Bone parameters also showed decreasing trends, however, they were not statistically significant (Fig. 2).

**FIG. 3.**
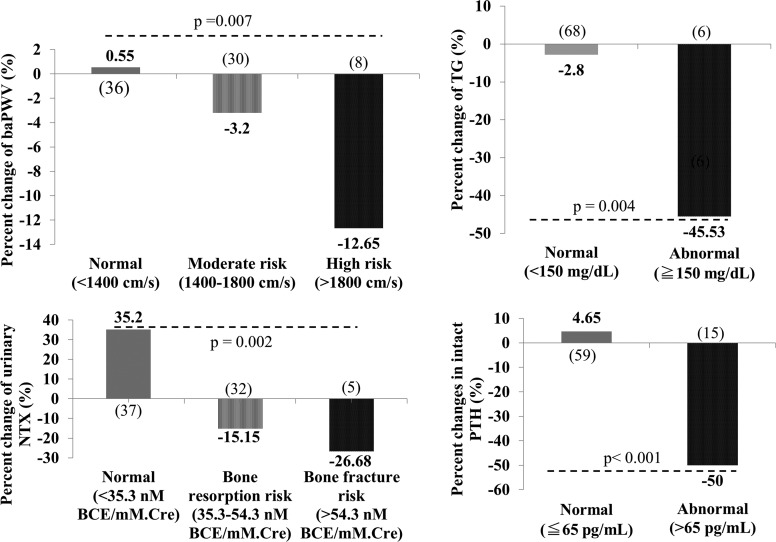
Post-treatment changes in parameters with respect to their initial risk categories. People in the high risk categories showed reduction in the above parameters. The values were expressed in medians, and *p*-values were assessed by Kruskal–Wallis tests. (), number of patients; baPWV, branchial-ankle pulse wave velocity; NTX, N-telopeptide; PTH, parathyroid hormone; TG, triglycerides.

As shown in Figure 3, significant reductions in baPWV were noted in patients with moderate arteriosclerotic risk (baPWV 1400–1800 cm/s, *n* = 30) and in high arteriosclerotic risk (baPWV ≧1800 cm/s, *n* = 8). In addition, significant reductions in parameters were observed in patients with hypertriglyceridemia (TG ≧150 mg/dL, *n* = 6), those with high risk for bone resorption (NTX 35.3–54.3 nmol/L BCE/mM.Cre, *n* = 32) or fracture (NTX >54.3 nmol/L BCE/mM.Cre, *n* = 5), and all the women with abnormal parathyroid hormone levels (PTH >65 pg/mL, *n* = 15) at the baseline.

As shown in Table 2, these initial risk categories were still the main predictors of percent decline in the parameter levels, except for baPWV, in the multiple regression analyses. In addition, there was a negative association with the participant's age and percent change in TG level after 12 months. In other words, younger women could be benefitted from reduction in TG levels after 12 months. Equol producers had a tendency to have reduced urinary NTX levels compared to equol nonproducers; however, this association missed a statistical significance.

**Table 2.** Summary of Multiple Regression Analyses (*n* = 72)

		*95% CI*		
*Variable*	B	*Lower limit*	*Upper limit*	p
Dependent variable: percent changes in baPWV				
Initial baPWV risk group	−5.440	−11.374	0.494	0.072
Age	0.221	−0.268	0.710	0.371
HRT treatment	−4.477	−11.187	2.232	0.187
Equol producer	2.284	−4.198	8.766	0.484
Lifestyle-related disease treatment	3.618	−2.879	10.116	0.270
Dependent variable: percent changes in TG				
Initial TG risk group	−50.345	−80.625	−20.064	**0.001**
Age	1.162	0.072	2.252	**0.037**
HRT treatment	0.747	−16.915	18.410	0.933
Equol producer	4.799	−12.067	21.664	0.572
Lifestyle-related disease treatment	6.146	−10.490	22.783	0.463
Dependent variable: percent changes in urinary NTX				
Initial urinary NTX risk group	−45.188	−68.337	−22.039	**<0.001**
Age	−0.635	−2.560	1.290	0.512
HRT treatment	−24.134	−54.358	6.090	0.116
Equol producer	−27.642	−56.310	1.025	0.059
Osteoporosis treatment	−5.454	−40.079	29.172	0.754
Dependent variable: percent changes in PTH				
Initial PTH risk group	−51.691	−75.118	−28.265	**<0.001**
Age	0.392	−0.912	1.697	0.550
HRT treatment	−3.053	−23.557	17.451	0.767
Equol producer	−13.463	−33.147	6.221	0.177
Osteoporosis treatment	−8.160	−31.343	15.023	0.485

*p* < 0.05 were bold.

B, unstandardized regression coefficient; baPWV, branchial-ankle pulse wave velocity; CI, confidence interval (of partial correlation coefficients); HRT, hormone replacement therapy; NTX, N-telopeptide; PTH, parathyroid hormone; TG, triglyceride.

### Safety

There were no reported adverse events or abnormal findings in blood cells, liver and kidney function tests, at periodic follow-ups. Transvaginal ultrasound for endometrial thickness, Pap smear, and mammography showed no significant changes after 12 months. In addition, no significant changes were noted in underlying gynecological conditions such as uterine fibroids.

## Discussion

Equol-producer status has been associated with decreased blood pressure and improved arterial stiffness and endothelial function^30,33,34^ Being an equol producer has been associated with reduced risk for coronary calcification in Japanese male adults.^35^ We have observed favorable cardiovascular effects such as reduced baPWV, homocysteine, high-sensitivity C-reactive protein, and high eicosapentanoic acid to arachidonic acid ratio in equol-producing women around 50 and 60 years of age.^10^ The baseline median baPWV value was lower in the equol producer group in this study but it was not statistically significant, which is contrary to our previous study. The reason for this discrepancy might be explained by being a small sample size and different target population. Our previous study found the association of equol-producer status with reduced baPWV among 214 women in the regular medical check-up who were relatively healthy. On the other hand, 74 women in this study came to the clinic for postmenopausal or certain medical complaints, where naturally occurring equol might be insufficient for body homeostasis. The same reason could be considered for nonsignificant results between equol producers and nonproducers in lipid and bone parameters at the baseline.

The effect of equol supplementation on arterial stiffness was reported in some short-term clinical studies.^28,30^ Equol significantly induced reduction in baPWV as early as 3 months after supplementation, compared with HRT, in our unpublished small clinical study.^*^ This is the first study in which a significant reduction in arterial stiffness was reported after 12-month supplementation with equol. The reduction in baPWV was more prominent among those with high baseline risks for arteriosclerosis, who had baPWV more than 1400 cm/s at the baseline. The mechanism of equol actions on arteriosclerosis might be not only via conventional estrogen receptors but also through the production of vasodilators such as nitric oxide.^36,37^

Regarding blood lipids, equol producers tend to have better improvements compared to nonproducers,^10,38,39^ and therefore, equol supplementation is expected to positively affect lipid levels. The mechanism of equol action on blood lipids might be owing to increased insulin sensitivity through estrogen receptor beta (ERβ)-mediated regulation of lipogenesis and glucose metabolism, similar to intrinsic estrogen.^27,39^ In this study, we observed that good cholesterol (HDL) levels were increased after equol treatment; however, the bad cholesterol level had also increased and the index for arteriosclerosis (LDL/HDL ratio) showed no significant change. These results were hard to discern and further well-designed clinical trials are warranted to examine the effects of equol on blood cholesterol level. It is also interesting that, although the decline in TG levels was not significant in all participants, women with hypertriglyceridemia (>150 mg/dL) were more likely to benefit from equol treatment, even after multivariate analysis including medication history.

Menopause is said to be associated with a rapid phase of bone loss, resulting from an imbalance between bone resorption and bone formation due to estrogen deficiency.^40^ Women who are equol producers showed slower bone density reduction compared with equol nonproducers^41,42^ and there was association of equol-producer status and reduction of bone resorption markers, such as urinary deoxypyridinoline^43^ and urinary NTX.^10^ Studies using animal cell lines have shown that equol inhibits osteoclast formation,^44^ a result that may be attributed to reduction in bone resorption markers. In this study, we found that women with the high baseline risk for bone resorption or fracture were more likely to have reduced urinary NTX levels after equol intervention; this is the first such finding among women in a clinical setting.

PTH levels were greatly decreased after the intervention in 15 women with abnormal PTH levels (PTH >65 pg/mL). To be exact, all those women achieved normal PTH levels, that is, below 65 pg/mL, after 12 months supplementation with equol. There are conflicting reports on the presence of estrogen receptors in parathyroid glands. However, the regulation of PTH is mediated mostly by fibroblast growth factor 23 (FGF23)^45^ and its expression has been shown to be under the influence of estrogen in rats.^46^ Additionally, estrogen actions on Ca, vitamin D, and phosphorous might indirectly affect the negative feedback regulation of PTH,^47^ and equol's estrogenic action might be responsible for reduction in intact PTH level. There have been no previous reports on the relationship between intact PTH and isoflavones; therefore, ours is the first study to report this association.

This study has several limitations. First, the change in food habits and supplement history were not assessed, and therefore, the outcomes of the study might have been affected by the type of diets such as isoflavones or prebiotics and other relevant food habits.^48,49^ However, diet is not the sole contributor for gut environment and equol metabolism or actions. For instance, host genetic variants might also be responsible for variations in equol metabolism and bioavailability.^7,9^ Also, it takes time to convert an equol nonproducer to a producer. Even for the producers to consistently maintain the efficacy of equol, that is, to maintain the blood level of biologically available equol, they need to consume at least 50 mg of soy isoflavones daily, which is very difficult to achieve. In addition, women in this study were patients with postmenopausal or medical problems, who might require more potent dosage of equol rather than a trace amount of naturally occurring equol inside the body. For those reasons, we assumed that the effect of diet was modest compared to daily equol supplementation in these women.

Second, our study outcomes had mainly focused on the biomarkers rather than the patient-centered outcomes. However, with the improvements seen in baPWV (arterial stiffness), we have clinically observed that most patients enjoyed better blood pressure control and relief in vasomotor symptoms such as hot flashes along with the stable blood pressure. In fact, we also noticed significant improvements in the perceived climacteric symptoms, assessed in the monthly questionnaires. The improvements were observed in both groups taking equol alone and combination with HRT, regardless of equol-producing ability (Supplementary Data; Supplementary Data are available online at www.liebertpub.com/acm). However, those results should be taken with caution as there was no placebo group and we could not definitely exclude the improvements as a result of time.

Third, we could not confirm the 100% adherence to equol supplementation in all the women who have completed the 12-month follow-up. Only 80% of them (60 out of 74 women) returned the monthly questionnaires, noting that they had been taking the supplement almost every day. Fourth, there was no comparison with a placebo group and sample size was limited as this was a prospective, observational study. Therefore, there is a possibility that the efficacy and safety of equol supplementation might have been missed due to potential type II error. Larger confirmatory studies are needed to warrant the findings.

Despite these limitations, our study is, notably, the first to examine the effects of 1-year supplementation, especially on bone parameters, and tolerability using follow-up screening for cervical and breast cancers in addition to endometrial conditions. Since this study could provide valuable information on both efficacy and safety, it could be useful as a reference study for those who want to try equol as an alternative or complementary therapy for the wellbeing of middle-aged women in real clinical setting.

## Conclusion

This study suggested that reduction in arterial stiffness was possible after 12 month supplementation with equol. Especially, positive responses could be seen in women in the high-risk groups such as those with hyperglycemia, high risk for arteriosclerosis, bone resorption and fracture, and abnormal PTH levels. These positive effects of equol alone or in combination with HRT suggest that equol might behave in a manner similar to selective estrogen receptor modulators. It might serve as a new, promising, and safe therapeutic option to offer as an alternative or complementary therapy in addition to proven therapies for women affected by estrogen deficiency. In addition, we observed that 1-year equol supplementation was well tolerated without any adverse events. Future well-designed randomized controlled trials are needed to validate the long-term use of equol for customized care of women, especially in those in the late reproductive life who are starting to be affected by low levels of intrinsic estrogen.

## Supplementary Material

Supplemental data

## Notes

^*^ Yoshikata R, Ohta H, Myint K. Comparison of equol containing dietary supplement with hormone replacement therapy in the improvement of vasomotor symptoms and secondary vascular actions in women with climacteric symptoms. Poster presented at the annual meeting of the North American Menopause Society, Las Vegas, NV, October 2015. Abs
