# Supplementary figures and images for: Effects of Equol Supplement on Bone and Cardiovascular Parameters in Middle-Aged Japanese Women: A Prospective Observational Study
Source: J Altern Complement Med. 2018 Jul 1;24(7):701–8. doi: 10.1089/acm.2018.0050 (PMC6065522; doi:10.1089/acm.2018.0050)

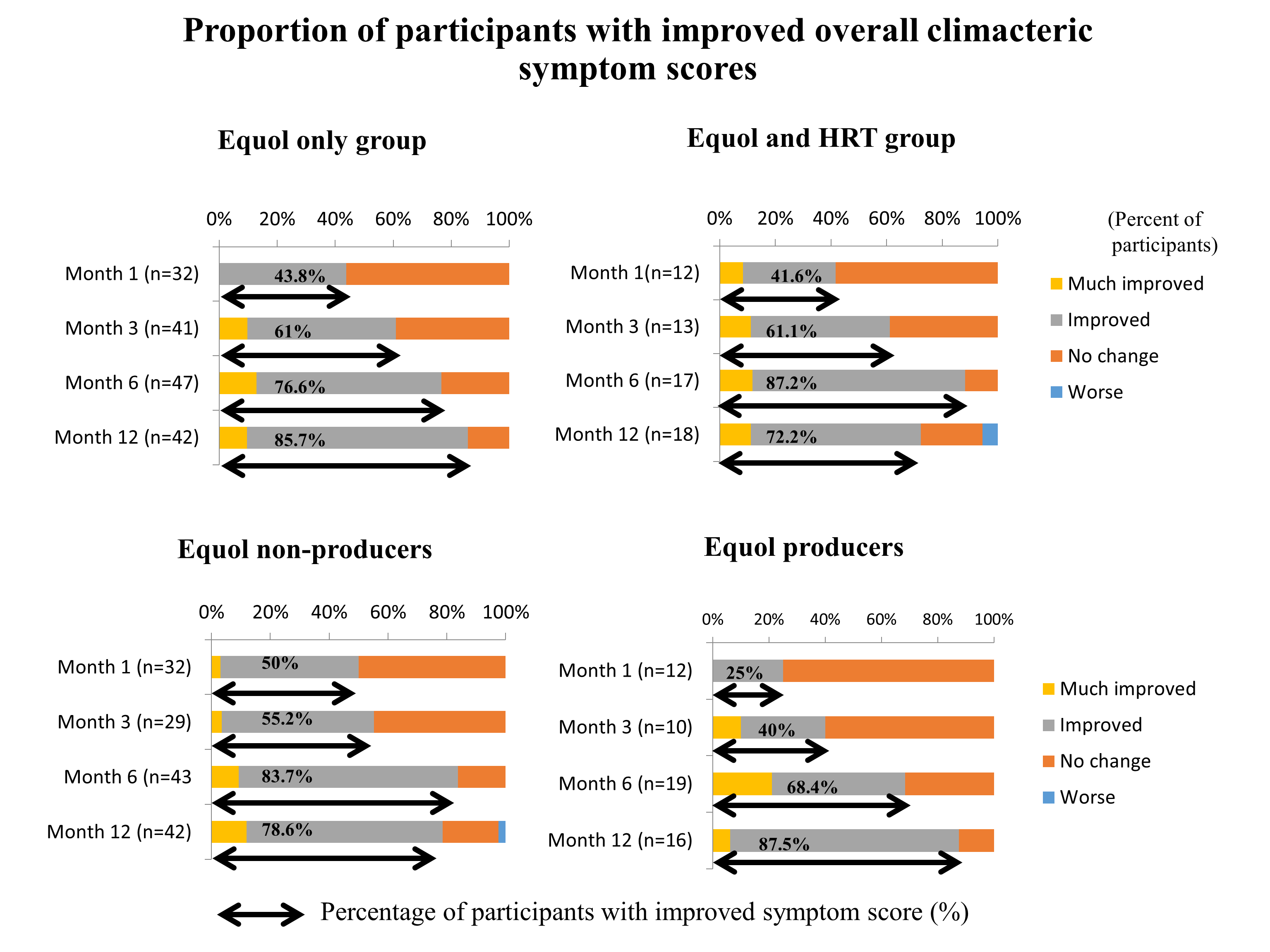

Supplement: Supplemental data [file Supp_Data.tif]
